# Supplementary material for: Individual and school-level factors associated with suspected pediatric eye disorders and referral adherence in an enhanced school-based vision screening program in Ghana
Source: PLOS Glob Public Health. 2026 Jun 3;6(6):e0006000. doi: 10.1371/journal.pgph.0006000 (PMC13232807; doi:10.1371/journal.pgph.0006000)
Supplement: S1 Text — (DOCX) [file pgph.0006000.s001.docx]

S1 Text. Description of the Enhanced Vision Screening Protocol

The enhanced vision screening protocol was implemented among consented students on the school premises from 4th to 8th November 2024 at the public school, and from 25th November to 4th December 2024 at the private school. Demographic data (e.g., sex, age) were extracted from school enrollment records where available, as well as during in-person interviews with children. Interviews were developed for this study (supplementary materials).

This study specifically adopted a simplified, economical approach tailored to a low-resource setting. By aligning with the practical constraints and priorities of such environments, the protocol aims to enhance its relevance and appeal to national policymakers and decision-makers. Insights from this pilot implementation will serve as a foundation for strategic refinement of the program over time. The vision screening protocol was described as ‘enhanced’ because vision screening was performed by local eye care professionals (ECPs), i.e., 10 optometrists and four optometry students affiliated with the Department of Optometry and Visual Science at the Kwame Nkrumah University of Science and Technology. Also, the protocol included direct ophthalmoscopy as part of vision screening. Screeners received prior training in vision screening procedures and obtaining case histories in line with the protocol.

Distance visual acuity for the right, left, and both eyes was obtained with and without existing prescription using the Lea symbols in a well-illuminated room measuring 6 meters (20 feet) distance. Near visual acuity was tested at the participants’ habitual reading distance. Pinhole examination was performed for participants whose visual acuities were 20/40 or worse for children aged 4 years, and 20/30 or worse for children older than 4 years. Estimates of refractive errors were obtained with a photoscreener (PlusoptiX S12C, software version 8.0.3.0; plusoptiX GmbH, Nuremberg, Germany) in a separate room with less illumination. A pen light was used to assess the external eye structures and anterior segment, while a direct ophthalmoscope was used to examine the optic nerve and macula of both eyes through an undilated pupil. In this study, an ocular disorder was defined as the presence of visually significant refractive error cases, and ocular anomalies identified in the screening using an enhanced screening protocol. Unlike comprehensive eye exams, vision screenings are limited to identifying potential ocular disorders without providing definitive diagnoses, as they do not assess overall ocular health.^18^ Therefore, all cases identified in this pilot study were considered "suspected" and required further comprehensive examination for diagnosis.

Due to the lack of standard national or international criteria informing decisions to refer, referrals from screening were provided in line with criteria developed by the American Academy of Pediatrics, the National Center for Children’s Vision and Eye Health (NCCVEH) (45), and the American Academy of Pediatric Ophthalmology and Strabismus.^19,20^ The criteria included age-based referral criteria for photoscreening, visual acuity testing and physical or functional vision screening tests (see S1 Table). Children were also referred if at least a two-line difference in visual acuity (VA) was observed between the two eyes, even when both eyes had VA within normal ranges. In addition, children were referred if they could not complete any of the screening tests after two attempts, including cases of malfunction of instrument-based cameras or complaints about their eyes or vision. Given the high prevalence of glaucoma in Ghana,^21,22^ even among children and young adults, the research team also referred children with a suspicious disc defined as a Cup to Disk Ratio ≥ 0.5, or a CDR difference of 0.2 between each eye, or the presence of a laminar dot sign.
