# Supplementary material for: Individual and school-level factors associated with suspected pediatric eye disorders and referral adherence in an enhanced school-based vision screening program in Ghana
Source: PLOS Glob Public Health. 2026 Jun 3;6(6):e0006000. doi: 10.1371/journal.pgph.0006000 (PMC13232807; doi:10.1371/journal.pgph.0006000)
Supplement: S1 Table — (DOCX) [file pgph.0006000.s002.docx]

S1 Table. Referral criteria for photoscreener and visual acuity testing in the study population

1. Age-based referral criteria used in the vision screening study

| ARF or Refractive Error | Age | Threshold |
| --- | --- | --- |
| ARF (severity ranked) |  |  |
| Media opacity | All ages | > 0.1mm |
| Strabismus | All ages | > 8 PD manifest |
| Anisometropia | All ages | > 1.25 D |
| Hyperopia | All ages | > 4.00 D |
| Astigmatism | < 4 years | > 3.00 D |
| Visually Significant Refractive Errors |  |  |
| Astigmatism | ≥ 4 years | > 1.75 D |
| Myopia | < 4 years | < -3.00 D |
| Myopia | ≥ 4 years | < -2.00 D |

Abbreviations: ARF, amblyopia risk factor; D, diopters; PD, prism diopters.
Note. American Academy of Pediatric Ophthalmology and Strabismus (AAPOS) uniform guidelines for instrument-based pediatric vision screen validation 2021 by Arnold et al.^1^

1. Age-dependent critical line for visual acuity testing

| Age (months) | Visual Acuity Critical Line to Pass |
| --- | --- |
| 36 – 47 | 20/50 |
| 48-59 | 20/40 |
| ≥60 | 20/30 (or 20/32 line on some charts) |

Note. “Procedures for the Evaluation of the Visual System by Pediatricians” 2016 by Donahue et al. and Pang et al.. ^2,3^

**Children who could not complete any of the screening tests after two screening attempts or with a two-line difference in visual acuity between the eyes were also referred.**

1. Arnold RW, Donahue SP, Silbert DI, et al. AAPOS uniform guidelines for instrument-based pediatric vision screen validation 2021. *J AAPOS*. 2022;26(1):1.e1–1.e6. doi:10.1016/j.jaapos.2021.09.009

2. Donahue SP, Baker CN, PRACTICE CO, et al. Procedures for the Evaluation of the Visual System by Pediatricians. *Pediatrics*. 2016;137(1)doi:10.1542/peds.2015-3597

3. Pang Y, Lyons SA, Nottingham Chaplin PK, Block SS, Fishman D, Ciner EB. Recommended practices for vision screening in pre-school-age children: A 2025 update. *Optom Vis Sci*. Oct 2025;102(10):589–95. doi:10.1097/opx.0000000000002290
