# Supplementary material for: Individual and school-level factors associated with suspected pediatric eye disorders and referral adherence in an enhanced school-based vision screening program in Ghana
Source: PLOS Glob Public Health. 2026 Jun 3;6(6):e0006000. doi: 10.1371/journal.pgph.0006000 (PMC13232807; doi:10.1371/journal.pgph.0006000)
Supplement: S3 Table — (DOCX) [file pgph.0006000.s004.docx]

S4 Table. Descriptive characteristics of children and the presence of suspected non-refractive errors

| **Characteristic** | **Total**  **(n=1,123)** | **Presence of Suspected N**on-**Refractive Error (n, %)** | | ***p*-value ^a^** |
| --- | --- | --- | --- | --- |
|  |  | **Yes**  **(n=244)** | **No**  **(n=879)** |  |
| School Type  Public  Private | 437 (38.91)  686 (61.09) | 86 (35.25)  158 (64.75) | 351 (39.93)  528 (60.07) | **.184** |

Suspected non-refractive eye disorders were categorized based on structure and function. ^a^ *P-value based on Pearson Chi-square test*
