# Supplementary material for: Individual and school-level factors associated with suspected pediatric eye disorders and referral adherence in an enhanced school-based vision screening program in Ghana
Source: PLOS Glob Public Health. 2026 Jun 3;6(6):e0006000. doi: 10.1371/journal.pgph.0006000 (PMC13232807; doi:10.1371/journal.pgph.0006000)
Supplement: S4 Table — (DOCX) [file pgph.0006000.s005.docx]

S5 Table. Descriptive characteristics of children and the presence of suspected myopia

| **Characteristic** | **Total**  **(n=1,123)** | **Presence of Suspected Myopia (n, %)** | | ***p*-value ^a^** |
| --- | --- | --- | --- | --- |
|  |  | **Yes**  **(n=26)** | **No**  **(n=1,097)** |  |
| School Type  Public  Private | 437 (38.91)  686 (61.09) | 2 (7.69)  24 (92.31) | 435 (39.65)  662 (60.35) | **<.001** |

**^a^***P-value based on Fisher’s exact test because expected cell counts < 5*
