# Supplementary material for: Individual and school-level factors associated with suspected pediatric eye disorders and referral adherence in an enhanced school-based vision screening program in Ghana
Source: PLOS Glob Public Health. 2026 Jun 3;6(6):e0006000. doi: 10.1371/journal.pgph.0006000 (PMC13232807; doi:10.1371/journal.pgph.0006000)
Supplement: S6 Table — (DOCX) [file pgph.0006000.s007.docx]

S7 Table. Frequency and proportions of all suspected eye disorders detected by type of school

| Suspected Eye Disorders | Screened n (%)  (n= 1,123) | **Type of School n (%)** | | ***p-*value** ^b^ |
| --- | --- | --- | --- | --- |
|  |  | Public  (n= 437) | Private  (n=686) |  |
| Non-refractive ^a^ |  |  |  |  |
| Ocular adnexa |  |  |  |  |
| Yes | 10 | 2 (20.00) | 8 (80.00) | .332 |
| No | 1,113 | 435 (39.08) | 678 (60.92) |  |
| Anterior Segment |  |  |  | .679 |
| Yes | 25 | 11 (44.00) | 14 (56.00) |  |
| No | 1,098 | 426 (38.80) | 672 (61.20) |  |
| Posterior Segment |  |  |  | .183 |
| Yes | 210 | 73 (34.76) | 137 (65.24) |  |
| No | 913 | 364 (39.87) | 549 (60.13) |  |
| Ocular Motility |  |  |  | .389 |
| Yes | 1 | 1 (100.00) | 0 (0.00) |  |
| No | 1,122 | 436 (38.86) | 686 (61.14) |  |
| Ocular Alignment |  |  |  |  |
| Yes | 6 | 2 (33.33) | 4 (66.67) | 1.000 |
| No | 1,117 | 435 (38.94) | 682 (61.06) |  |
| Refractive |  |  |  |  |
| Myopia |  |  |  |  |
| Yes | 26 | 2 (7.69) | 24 (92.31) | <.001 |
| No | 1,097 | 435 (39.65) | 662 (60.35) |  |
| Hyperopia |  |  |  |  |
| Yes | 15 | 2 (13.33) | 13 (86.67) | .0059 |
| No | 1,108 | 435 (39.26) | 673 (60.74) |  |
| Astigmatism |  |  |  |  |
| Yes | 82 | 17 (20.73) | 65 (79.27) | <.001 |
| No | 1,041 | 420 (40.35) | 621 (59.65) |  |
| Anisometropia |  |  |  |  |
| Yes | 69 | 24 (34.78) | 45 (65.22) | .525 |
| No | 1,054 | 413 (39.18) | 641(60.82) |  |

Non-refractive^a^ eye disorders were categorized based on structure and function. Proportions represent row percentages, ^b^ p-values based on Fisher’s exact test
