# Supplementary material for: Individual and school-level factors associated with suspected pediatric eye disorders and referral adherence in an enhanced school-based vision screening program in Ghana
Source: PLOS Glob Public Health. 2026 Jun 3;6(6):e0006000. doi: 10.1371/journal.pgph.0006000 (PMC13232807; doi:10.1371/journal.pgph.0006000)
Supplement: S7 Table — (DOCX) [file pgph.0006000.s008.docx]

S8 Table. Frequency and proportions of suspected non-refractive and refractive eye disorders by referral adherence after an abnormal vision screening test results

|  | **Referred n (%)**  **(n= 299)** | **Referral Adherence n (%)** | | ***p-*value ^b^** |
| --- | --- | --- | --- | --- |
|  |  | **Yes (n= 98)** | **No (n= 201)** |  |
| **Refractive** |  |  |  | .209 |
| Yes | 119 (39.80) | 44 (44.90) | 75 (37.31) |  |
| No | 180 (60.20) | 54 (55.10) | 126 (62.69) |  |
| **Non-refractive** ^a^ |  |  |  | .600 |
| Yes | 189 (63.21) | 64 (65.31) | 125 (62.19) |  |
| No | 110 (36.79) | 34 (34.69) | 76 (37.81) |  |

^a^ Non-refractive eye disorders were categorized based on structure and function. **^b^** *P-value based on Pearson Chi-square test*
