# Supplementary material for: Individual and school-level factors associated with suspected pediatric eye disorders and referral adherence in an enhanced school-based vision screening program in Ghana
Source: PLOS Glob Public Health. 2026 Jun 3;6(6):e0006000. doi: 10.1371/journal.pgph.0006000 (PMC13232807; doi:10.1371/journal.pgph.0006000)
Supplement: S8 Table — (DOCX) [file pgph.0006000.s009.docx]

S9 Table. Frequency and proportions of suspected non-refractive and refractive eye disorders by referral adherence after an abnormal vision screening test results

| Suspected Eye Disorders | Referred n (%)  (n= 299) | **Referral Adherence (%)** | | ***p-*value^a^** |
| --- | --- | --- | --- | --- |
|  |  | Yes  (n= 98) | No  (n= 201) |  |
| Non-refractive |  |  |  |  |
| Ocular adnexa |  |  |  |  |
| Yes | 8 | 3 (37.50) | 5 (62.50) | 0.720 |
| No | 291 | 95 (32.65) | 196 (67.35) |  |
| Anterior Segment |  |  |  |  |
| Yes | 14 | 6 (42.86) | 8 (57.14) | 0.398 |
| No | 285 | 92 (32.28) | 193 (67.72) |  |
| Posterior Segment |  |  |  |  |
| Yes | 169 | 57 (33.73) | 112 (66.27) | 0.711 |
| No | 130 | 41 (31.54) | 89 (68.46) |  |
| Ocular Motility |  |  |  |  |
| Yes | - | - | - | - |
| No | 299 | 98 (32.78) | 201 (67.22) |  |
| Ocular Alignment |  |  |  |  |
| Yes | 5 | 2 (40.00) | 3 (60.00) | 0.664 |
| No | 294 | 96 (32.65) | 198 (67.35) |  |
| Refractive |  |  |  |  |
| Myopia |  |  |  |  |
| Yes | 17 | 6 (35.29) | 11 (64.71) | 0.796 |
| No | 282 | 92 (32.62) | 190 (67.38) |  |
| Hyperopia |  |  |  |  |
| Yes | 10 | 6 (60.00) | 4 (40.00) | 0.085 |
| No | 289 | 92 (31.83) | 197 (68.17) |  |
| Astigmatism |  |  |  |  |
| Yes | 58 | 20 (34.48) | 38 (65.52) | 0.757 |
| No | 241 | 78 (32.37) | 163 (67.63) |  |
| Anisometropia |  |  |  |  |
| Yes | 57 | 21 (36.84) | 36 (63.16) | 0.531 |
| No | 242 | 77 (31.82) | 165 (68.18) |  |

^a^ p-values based on Fisher’s exact test
