# Supplementary material for: Individual and school-level factors associated with suspected pediatric eye disorders and referral adherence in an enhanced school-based vision screening program in Ghana
Source: PLOS Glob Public Health. 2026 Jun 3;6(6):e0006000. doi: 10.1371/journal.pgph.0006000 (PMC13232807; doi:10.1371/journal.pgph.0006000)
Supplement: S9 Table — (DOCX) [file pgph.0006000.s010.docx]

S10 Table. Association between exposure variables and the presence of suspected refractive error detected in the vision screening study

| **Exposure** | **Presence of a Suspected Refractive Error Disorders** | | | | |
| --- | --- | --- | --- | --- | --- |
|  | **OR (95%)** | ***p*-value** | **aOR (95%)** | ***p*-value** |  |
| School Type  Public  Private | 0.42 (0.29, 0.63)  Reference | <0.001  - | 0.38 (0.21, 0.68)  Reference | 0.001  - |  |
| Socioeconomic Status  Low  Moderate  High | 0.58 (0.35, 0.95)  1.40 (0.90, 2.17)  Reference | 0.032  0.141  - | 1.17 (0.62, 2.23)  1.57 (1.00, 2.47)  Reference | 0.623  0.049  - |  |
| Sex  Male  Female | 1.35 (0.96, 1.90)  Reference | 0.081  - | 1.26 (0.86, 1.84)  Reference | 0.234  - |  |
| Age, years | 1.00 (0.93,1.06) | 0.893 | 1.07 (0.99, 1.15) | 0.090 |  |

OR = odds ratio; aOR = adjusted odds ratio
