# Supplementary material for: Individual and school-level factors associated with suspected pediatric eye disorders and referral adherence in an enhanced school-based vision screening program in Ghana
Source: PLOS Glob Public Health. 2026 Jun 3;6(6):e0006000. doi: 10.1371/journal.pgph.0006000 (PMC13232807; doi:10.1371/journal.pgph.0006000)
Supplement: S10 Table — (DOCX) [file pgph.0006000.s011.docx]

S11 Table. Association between exposure variables and the presence of suspected non-refractive error detected in the vision screening study

| **Exposure** | **Presence of a Suspected N**on-**Refractive Eye Disorders** | | | | |
| --- | --- | --- | --- | --- | --- |
|  | **OR (95%)** | ***p*-value** | **aOR (95%)** | ***p*-value** |  |
| School Type  Public  Private | 0.82 (0.61, 1.10)  Reference | 0.184  - | 0.91 (0.57, 1.45)  Reference | 0.697  - |  |
| Socioeconomic Status  Low  Moderate  High | 0.85 (0.58, 1.24)  0.85 (0.58, 1.26)  Reference | 0.397  0.424  - | 0.91 (0.54, 1.53)  0.86 (0.58, 1.29)  Reference | 0.715  0.475  - |  |
| Sex  Male  Female | 1.46 (1.10, 1.94)  Reference | 0.009  - | 1.45 (1.06, 1.98)  Reference | 0.021  - |  |
| Age, years | 1.00 (0.95,1.06) | 0.940 | 1.03 (0.97,1.09) | 0.380 |  |

OR = odds ratio; aOR = adjusted odds ratio
